# A metagenomic analysis coupled with oligotrophic enrichment approach for detecting specified microorganisms in potable groundwater samples

**DOI:** 10.3389/fmicb.2025.1645324

**Published:** 2025-08-13

**Authors:** Soumana Daddy Gaoh, Pierre Alusta, Yong-Jin Lee, David Hussong, Bernard Marasa, Youngbeom Ahn

**Affiliations:** ^1^Division of Microbiology, National Center for Toxicological Research, U.S. Food and Drug Administration, Jefferson, AR, United States; ^2^Division of Systems Biology, National Center for Toxicological Research, U.S. Food and Drug Administration, Jefferson, AR, United States; ^3^Department of Natural Sciences, Albany State University, Albany, GA, United States; ^4^Eagle Analytical Services, Houston, TX, United States; ^5^Office of Pharmaceutical Quality, Center for Drug Evaluation and Research, U.S. Food and Drug Administration, Silver Spring, MD, United States

**Keywords:** metagenomic analysis, oligotrophic enrichment, specified microorganisms, groundwater, non-sterile pharmaceutical product

## Abstract

In pharmaceutical manufacturing, there is a significant need for the detection and identification of specified microorganisms (*i.e.*, *Burkholderia cepacia* complex (BCC), *E. coli, Pseudomonas aeruginosa*, *Salmonella enterica, Staphylococcus aureus, Clostridium sporogenes, Candida albicans,* and *Mycoplasma*), which are often missed or not identified by traditional culture-dependent methods. We employed a metagenomic analysis coupled with oligotrophic enrichment to identify specified microorganisms and evaluate tryptic soy broth (TSB) and 1/10 strength TSB for the recovery of specific microorganisms in potable groundwater samples. A total of 589–996 genera were identified in 12 water samples taken from a cold water fountain, with *Bacillus* spp. (97%) in TSB and *Stenotrophomonas* spp. (97%) in 1/10 strength TSB, representing the primary recovered genera after a 72-h pre-enrichment at 23°C. Likewise, we also detected lower abundance of specific organisms, *Clostridium* spp., *Burkholderia* spp., and *Staphylococcus* spp. (0.04–0.07%) in TSB and *Burkholderia* spp., *Pseudomonas* spp., *Salmonella* spp., *Staphylococcus* spp. and *Escherichia* spp. (0.01–1.73%) in 1/10 strength TSB. Co-inoculation with *Burkholderia cepacia* complex (BCC) yielded a higher recovery rate of *Pseudomonas* spp. compared to uninoculated controls in 1/10 strength TSB. Further functional analyses indicated that, toluene degradation (PWY-5180 and PWY-5182) was chiefly contributed by BCC in co-cultures of TSB + BCC-24 h and TSB + BCC-48 h. Our results demonstrate the potential value of the metagenomic approach during enrichment in detecting specified microorganisms, including oligotrophs such as BCC in non-sterile pharmaceutical products.

## 1 Introduction

Non-sterile water-based drug and non-drug products have been shown to be contaminated with objectionable pathogens and have caused product recalls within the US. A report published in 2019 surveyed FDA recalls from 2012 to 2019, showed that *Burkholderia* spp. were the number one reason for non-sterile drug recalls (105 recalls) followed by *Ralstonia pickettii* (45 recalls) and *Salmonella* spp. (28 recalls) (Jimenez, 2019). Unidentified microbial contamination accounted for 77% of non-sterile and 87% of sterile drug recalls, indicating extremely poor microbiology practices. Overall, these pioneering surveillance reports clearly showed that a significant proportion of microbial contaminants was left unidentified. Water serves as the primary ingredient in pharmaceutical products and represents a significant source of microbiological contamination, as microorganisms, particularly Gram-negative bacteria, can proliferate in aquatic environments even with minimal nutrient availability. This inherent risk necessitates stringent quality control measures for testing water and liquid products used in pharmaceutical manufacturing, given that water presence alone creates substantial potential for microbial growth. According to FDA Inspection Technical Guides for “Water for Pharmaceutical Use” (FDA, 1986), various water types including non-potable, potable, purified, and high-purity water are utilized in pharmaceutical manufacturing, each posing serious microbiological contamination risks to final products, particularly when proper testing procedures are not implemented. The presence of certain microorganisms in non-sterile preparations may not only have the potential to reduce or even inactivate the therapeutic activity of drug products but also consequently adversely affect patient health. U.S. Pharmacopeia (USP) <1111> recommends acceptance criteria for the presence of certain microorganisms in non-sterile preparations based on the route of administration (USP1111, 2016). Furthermore, USP <60>, USP <61> and <62> testing is designed to demonstrate compliance with these requirements for the presence of specified microorganisms (*i.e.*, *E. coli, Pseudomonas aeruginosa*, *Salmonella* spp.*, Staphylococcus aureus, Clostridia* spp., *Candida albicans* and the *Enterobacteriaceae* family) (USP61, 2016; USP62, 2016)*, Burkholderia cepacia* complex (BCC) (USP60, 2018) and *Mycoplasma* (USP63, 2016). Although science has conclusively shown that the culture-based detection misses a great deal of microorganisms and investigations may be restricted unless a colony is recovered, culture-based methods (for sterility and microbial limits) are still the preferred Good Manufacturing Practices (GMPs). Additionally, USP has traditionally relied on the least common denominator for test methods (USP60, 2018; USP61, 2016).

Historically, investigations of microbial communities have relied on culture-based methodologies. However, less than 1% of bacterial species in environmental communities are thought to be culturable on standard laboratory growth media (Amann et al., 1995). Although BCC are able to grow and remain viable in hot or cold distilled water, most cells perish when transferred to Trypticase Soy Broth (TSB) medium (Carson et al., 1973). Recently, we recommended the use of oligotrophic media (1/10 strength Trypticase Soy Agar (TSA), 1/10 strength TSB, Reasoner’s 2nd Agar (R2A) or Reasoner’s 2nd Broth (R2AB)), which allow for improved recovery of BCC organisms present in distilled water or antiseptic samples (Ahn et al., 2019). Given the ability of BCC to evade detection, their aptitude to grow in low-nutrient conditions, and their resistance to antimicrobials and inherent pathogenic potential to especially immunocompromised individuals, new detection methods are required to ensure pharmaceutical product quality and patient safety. Culture-independent techniques such as PCR and real-time quantitative PCR (qPCR) assays led to a rapid and sensitive detection (Mahenthiralingam et al., 2000; Attia et al., 2016; Jimenez et al., 2018; Furlan et al., 2019; Tavares et al., 2020). We previously demonstrated the potential of droplet digital polymerase chain reaction (ddPCR), flow cytometry, loop-mediated isothermal amplification (LAMP), and recombinase polymerase amplification exo (RPA Exo) assay as more sensitive alternatives to culture-based methods to detect BCC in autoclaved nuclease-free water and antiseptic samples (Ahn et al., 2020; Daddy Gaoh et al., 2021; Daddy Gaoh et al., 2022a; Daddy Gaoh et al., 2022b; Daddy Gaoh et al., 2023). However, PCR should be coupled with a specific primer set designed to rapidly detect specific bacteria. To overcome this limitation, a molecular approach was suggested to characterize total microbial community DNA (including viruses, prokaryotes and eukaryotes). Consequently, next generation sequencing through shotgun metagenomic DNA sequencing was applied to assess the presence and the relative abundance of the microbiome of “specified microorganisms” (Daddy Gaoh et al., 2024). Metagenome-based approaches offer a more comprehensive view of the genetic complexity of natural and engineered microbial communities, allowing us to better assess the microbial taxonomic diversity and metabolic potential within any given community (Brumfield et al., 2020a; Thormar et al., 2024; Lin et al., 2025; Verma et al., 2025). The number of metagenomic studies has increased in recent years due to the availability of next generation sequencing technologies (Douterelo et al., 2018; Roy et al., 2018; Saleem et al., 2018; Brown et al., 2019; Farhat et al., 2019; Beraud-Martinez et al., 2020; Brumfield et al., 2020a; Brumfield et al., 2020b; Nam et al., 2023; Rout et al., 2023; Rout et al., 2024; Verma et al., 2025). It seems logical to be testing for the presence of genetic fingerprints before trying to culture the “specified microorganisms,” especially when they may not be culturable. A significant benefit of this approach is the ability to detect and identify specified microorganisms, including BCC in pharmaceutical manufacturing, which were previously missed or not identified by culture-dependent methods.

Our study mainly aims to detect specified microorganisms and BCC in enriched water samples. The objectives of this study are (i) to detect “specified microorganisms” (*i.e*., BCC (USP <60>), *E. coli, Pseudomonas aeruginosa*, *Salmonella enterica, Staphylococcus aureus, Clostridium sporogenes, Candida albicans* (USP <62>)*, Mycoplasma* (USP <63>)); (ii) to evaluate TSB and 1/10 strength TSB for the recovery of specified microorganisms; (iii) to understand the synergistic effects of BCC spiked for a better recovery of specified microorganisms; and (iv) to identify potential novel BCC functions encoded in BCC-spiked water.

## 2 Materials and methods

### 2.1 Experimental setup

**Figure 1:**
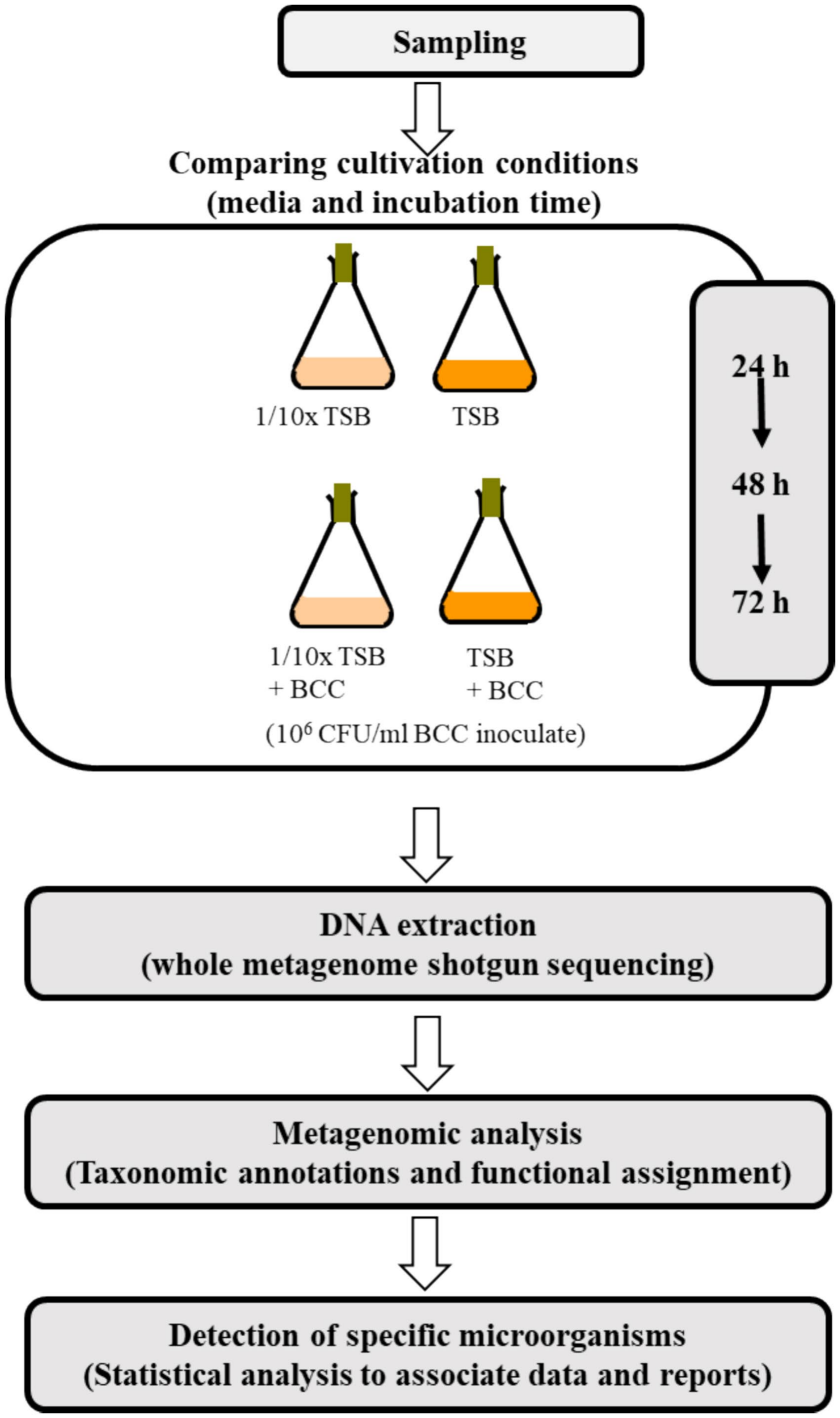
Overview of the metagenomics study.

The experimental conditions tested using cold water fountain are shown in Figure 1. To detect “specified microorganisms” and to evaluate TSB and 1/10 strength TSB for the recovery of specified microorganisms, 10 L of fresh water from a cold water fountain (potable-grade water) were collected from Hot Springs, AR (global positioning system (GPS) coordinates: Latitude: 34°51′52.83″, Longitude: −93°04′54.258″) in Sept. of 2021. Water samples were transported to the laboratory and processed within 1 h of collection to prevent microbial growth/decline. This source of water was chosen for its rich source of diverse flora, whereas pharmaceutical grade water contains far fewer species. Each 100 mL of the water sample were equally aliquoted to 4 (250 mL) flasks. To enrich the water samples with TSB media, 3 g of TSB were added to each 100 mL of water sample. To prepare 1/10 strength TSB (1/10 × TSB) media, 0.3 g of TSB powdered medium were added to each 100 mL of water sample, and incubated at 30°C under continuous agitation at 200 rpm for 72 h. For DNA extraction, a 1 mL sample was collected from each culture at 24 h, 48 h and 72 h after incubation. To understand the synergistic effects of BCC spiking for recovery of specified microorganisms, a fresh culture of *B. cenocepacia* AU1054 was inoculated into 100 mL TSB and 1/10 × TSB to approximately 10^6^ CFU/mL (Ahn et al., 2014; Ahn et al., 2019), then incubated at 30°C under continuous agitation at 200 rpm for 72 h.

### 2.2 Colony counts on 1/10 strength TSA and BCC-specific medium

To compare the recovery efficiency of TSB and 1/10 × TSB with/without spiked BCC, a 1 mL water sample from each of the four flasks (24 h, 48 h and 72 h) was used to prepare a 10-fold dilution series in autoclaved distilled water. As little as 10 μL of each serial dilution were plated onto prepared 1/10 strength TSA (1/10 × TSA) and BCC-specific medium (1/10 × TSA with vancomycin, gentamicin, and polymyxin B, Thermo Fisher Scientific, USA). Cultures were incubated for 48 h at 30°C and compared for their recovery in 1/10 × TSA and BCC specific medium.

### 2.3 DNA extraction and whole metagenome shotgun sequencing

One milliliter of each culture sample from each flask on culture days 1–3 were collected and centrifuged daily at 10,000 × g for 20 min. Total DNA was extracted from the pellet in triplicate using a Qiagen Kit according to the manufacturer’s instructions (Qiagen, Germantown, MD, USA). DNA quantification was measured using a NanoDrop ND-2000 spectrophotometer (Thermo Fisher Scientific Inc.). Furthermore, the initial concentration of DNA was evaluated using the Qubit® dsDNA HS Assay Kit (Life Technologies, Carlsbad, CA, USA). Due to an insufficient DNA concentration of the 24 h sample 1/10 × TSA, whole genome amplification was carried out using the REPLI-g Midi kit (Qiagen). The linear amplification of DNA was cleaned using the DNEasy PowerClean Pro Cleanup Kit (Qiagen) and concentrations were again evaluated (Table 1) using the Qubit® dsDNA HS Assay Kit (Life Technologies). Sequencing was performed with a NovaSeq 6000 instrument as per manufacturer’s instructions (Molecular Research LP, Shallowater, TX, USA) by MR DNA (Shallowater, TX, USA)^1^ (Daddy Gaoh et al., 2024). Libraries were prepared using Illumina DNA Prep, (M) Tagmentation library preparation kit (Illumina) following the manufacturer’s user guide. Briefly, 50 ng of DNA were used to prepare the libraries. The samples underwent simultaneous fragmentation and addition of adapter sequences. These adapters are utilized during a limited-cycle PCR in which unique indices were added to the sample. Following library preparation, the final concentrations of the libraries were measured using the Qubit® dsDNA HS Assay Kit (Life Technologies), and the average library size (Table 1) was determined using the Agilent 2,100 Bioanalyzer kit (Agilent Technologies). The libraries were then pooled in equimolar ratios of 0.7 nM and sequenced for 150 bp paired end reads with 300 cycles using the NovaSeq 6000 system (Illumina).

**Table 1:** Summary of samples including DNA and sequencing information of metagenomic analysis from cold fountain water samples.

Sample name	Medium	BCC added	Culture time	DNA conc. (ng/μl)	Final library DNA conc. (ng/μl)	Average Library size (bp)	Average GC content (%)	DNA paired end reads; Raw	Final sequencing libraries
TSB-24 h	TSB		24 h	169.2	23.40	657	35	26,421,188	9,240,862
TSB + BCC-24 h	TSB	BCC	24 h	412.0	16.10	742	66	28,641,055	23,660,634
1/10 × TSB-24 h*	1/10 × TSB		24 h	too low; 404.0**	23.40	744	49	21,103,554	9,529,702
1/10 × TSB + BCC-24 h	1/10 × TSB	BCC	24 h	432.0	19.30	648	66	34,121,260	28,925,622
TSB-48 h	TSB		48 h	212.0	21.20	703	34	24,216,049	8,481,220
TSB + BCC-48 h	TSB	BCC	48 h	928.0	22.20	713	66	25,418,445	22,075,138
1/10 × TSB-48 h	1/10 × TSB		48 h	940.0	19.70	697	66	29,205,073	22,207,514
1/10 × TSB + BCC-48 h	1/10 × TSB	BCC	48 h	624.0	18.60	636	66	40,029,112	34,094,584
TSB-72 h	TSB		72 h	230.0	20.40	713	34	18,508,113	7,052,140
TSB + BCC-72 h	TSB	BCC	72 h	1220.0	19.20	740	66	24,901,170	21,671,352
1/10 × TSB-72 h	1/10 × TSB		72 h	696.0	12.10	765	66	34,465,670	24,475,759
1/10 × TSB + BCC-72 h	1/10 × TSB	BCC	72 h	692.0	20.00	699	66	26,924,498	23,133,632

*1/10 × TSB: 1/10 strength. **After linear amplification of DNA.

### 2.4 Metagenomic analysis

The resulting sequences were uploaded to the Illumina server.^2^ The Nephele QC pipeline was used to run a quality control check (FastQC), trim primers and/or adapters, trim and/or filter reads based on quality scores, merge read pairs, and provide summary graphs of the QC steps (Bolger et al., 2014; Ewels et al., 2016). We used the bioBakery pipeline at Nephele^3^ to run whole metagenome sequence data analysis (McIver et al., 2018; Weber et al., 2018). Based on the best taxonomic assignment for the bacterial domain, considering *Escherichia coli, Pseudomonas aeruginosa*, *Salmonella* spp.*, Staphylococcus aureus, Clostridium* spp., *Candida albicans*, BCC and *Mycoplasma* spp., each sequence was classified into its genus level using Microsoft Excel. Significant differences between the detection of specific microorganisms obtained by TSB, 1/10 × TSB, TSB + BCC and incubation time were determined using SigmaPlot vs. 14.1 software.

## 3 Results

### 3.1 Viable bacterial counting

Without enrichment of water samples, 10 μL of cold fountain water were placed onto prepared TSA, 1/10 × TSA and BCC-specific medium. Both heterotrophic bacteria and BCC did not grow within 24 h on any of the tested agar media inoculated with cold fountain water. Interestingly, BCC did not grow on the BCC-specific medium even after 72 h of incubation at 30°C (data not shown).

**Figure 2:**
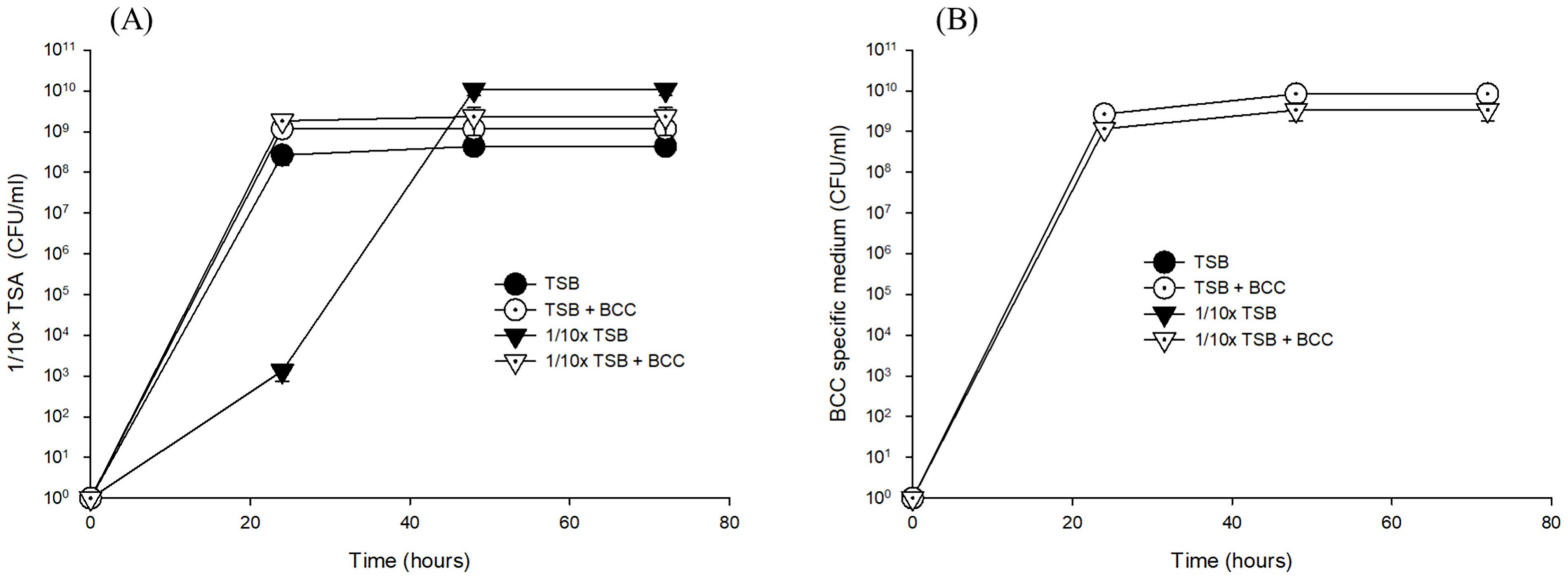
Growth of total heterotrophic bacteria on 1/10 strength TSA **(A)** and BCC on BCC-specific medium **(B)** on the corresponding agar media inoculated with cultures of pre-enriched cold fountain water using various media at 23°C over sampling time course.

However, after 24 h enrichment of water samples with TSB and 1/10 × TSB with/without spiked BCC (TSB, 1/10 × TSB, TSB + BCC and 1/10 × TSB + BCC), both heterotrophic bacteria and BCC grew on 1/10 × TSA and BCC-specific medium, respectively (Figure 2). In TSB-enriched samples, colonies appeared on average 4.00 × 10^8^ ± 1.73 × 10^8^ CFU/mL ~ 3.66 × 10^8^ ± 0.58 × 10^8^ CFU/mL over 72 h using 1/10 × TSA (Figure 2A). Furthermore, colony counts were approximately the same in TSB + BCC sample (1.57 × 10^9^ ± 0.42 × 10^9^ CFU/mL ~ 7.00 × 10^9^ ± 2.00 × 10^9^ CFU/mL), and 1/10 × TSB + BCC sample (2.67 × 10^9^ ± 0.58 × 10^9^ CFU/mL ~ 3.67 × 10^9^ ± 1.53 × 10^9^ CFU/mL) over 72 h. However, the average colony number of 2.00 × 10^3^ CFU/mL in 1/10 × TSB sample at 24 h was less than those of TSB, TSB + BCC and 1/10 × TSB + BCC samples at the same time period. After that, heterotrophic bacteria showed an increase in colony numbers until the 48-h period, and the counts remained stationary thereafter (9.33 × 10^9^ ± 2.89 × 10^9^ CFU/mL).

When it comes to detecting BCC in TSB and 1/10 × TSB enriched samples after 24, 48, and 72 h using BCC-specific media, no growth was detected, while in spiked samples no significant difference in growth between TSB + BCC sample (2.67 × 10^9^ ± 5.77 × 10^8^ CFU/mL ~ 8.33 × 10^9^ ± 3.06 × 10^9^ CFU/mL) and 1/10 × TSB + BCC sample (1.17 × 10^9^ ± 5.77 × 10^8^ CFU/mL ~ 3.33 × 10^9^ ± 1.53 × 10^9^ CFU/mL) was observed when cultivated in BCC-specific medium over 72 h (Figure 2B).

### 3.2 Detecting “specified microorganisms”

In this study, a total of 12 samples were used for sequencing. The description of each sample is provided in Table 1. Shotgun metagenomic sequencing, using total DNA prepared from the 12 samples, generated approximately an average of 2.78 × 10^7^ reads (Supplementary Figure S1). The sequencing libraries yielded an average of 1.95 × 10^7^ high-quality metagenomic sequences, with the number of sequencing reads ranged from 0.7 × 10^7^ reads in TSB after 72 h (TSB-72 h) to 3.4 × 10^7^ reads in 1/10 × TSB with BCC after 48 h (1/10 strength TSB + BCC-48 h) (Table 1; Supplementary Table S1). Despite insufficient DNA concentration from 1/10 strength TSB-24 h, the number of sequencing reads amounted to 0.9 × 10^7^ reads.

We observed a total of 589–996 bacterial genera in water collected from the cold water fountain. Dominant bacterial phyla detected were *Firmicutes* in TSB samples, *Gammaproteobacteria* in 1/10 strength TSB samples, and *Betaproteobacteria* in all BCC-spiked samples. In TSB and 1/10 × TSB samples, sequences were generally dominated by one genus. *Bacillus* spp. were dominant in TSB over a period of 72 h, at a 97–99% relative sequencing read abundance, and also detected in 1/10 × TSB over 24 h (29.4%) (Supplementary Figure S2). However, in 1/10 × TSB, *Stenotrophomonas* spp. accounted for 61.5% of the relative sequencing read abundance after 24 h of incubation. After 48 h and 72 h incubation in 1/10 × TSB, *Stenotrophomonas* spp. accounted for over 97%. *Burkholderia cenocepacia* accounted for 99% of the relative sequencing read abundance in all BCC-spiked media after 72 h.

#### 3.2.1 Comparing TSB and 1/10 × TSB media

**Figure 3:**
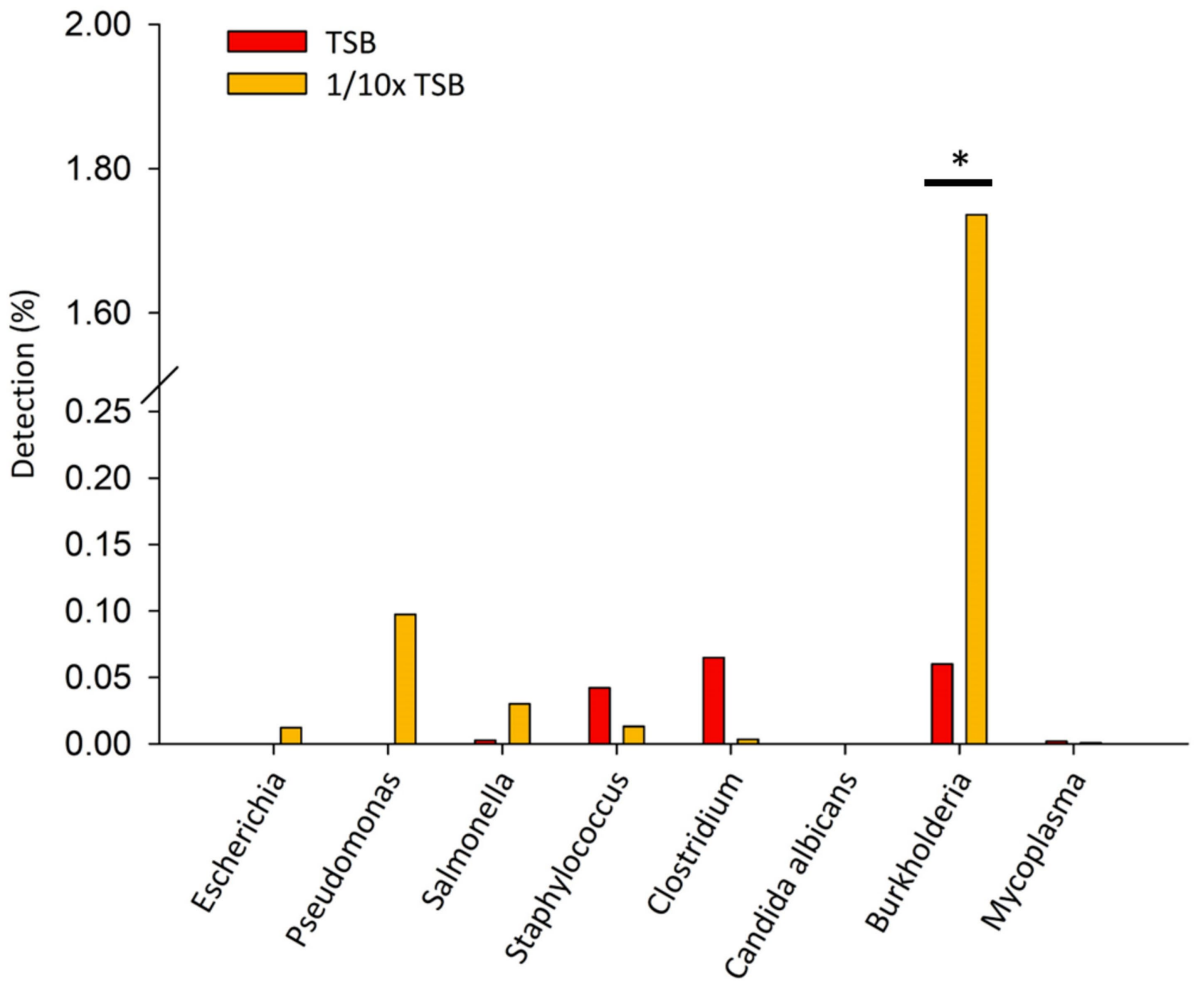
Detection of specified microorganism genera after 24 h enrichment in TSB and 1/10 strength TSB media. Results are expressed as relative sequence abundance of reads. The * symbol indicates a statistically significant difference, *p* < 0.05.

Specified microorganisms (*i.e.*, BCC, *E. coli, Pseudomonas aeruginosa*, *Salmonella enterica, Staphylococcus aureus, Clostridium sporogenes, Candida albicans,* and *Mycoplasma*) were observed less than 1.8% of relative sequencing read abundance in TSB and 1/10 × TSB after 24 h (Figure 3). *Candida albicans* was not detected. In TSB, *Clostridium* spp., *Burkholderia* spp., and *Staphylococcus* spp. were observed 0.07, 0.06, and 0.04%, respectively. In 1/10 × TSB, *Burkholderia* spp. (1.73%) was predominant in the samples. *Pseudomonas* spp., *Salmonella* spp., *Staphylococcus* spp. and *Escherichia* spp. were observed 0.09, 0.03, 0.01 and 0.01%, respectively.

#### 3.2.2 Comparing incubation times

**Figure 4:**
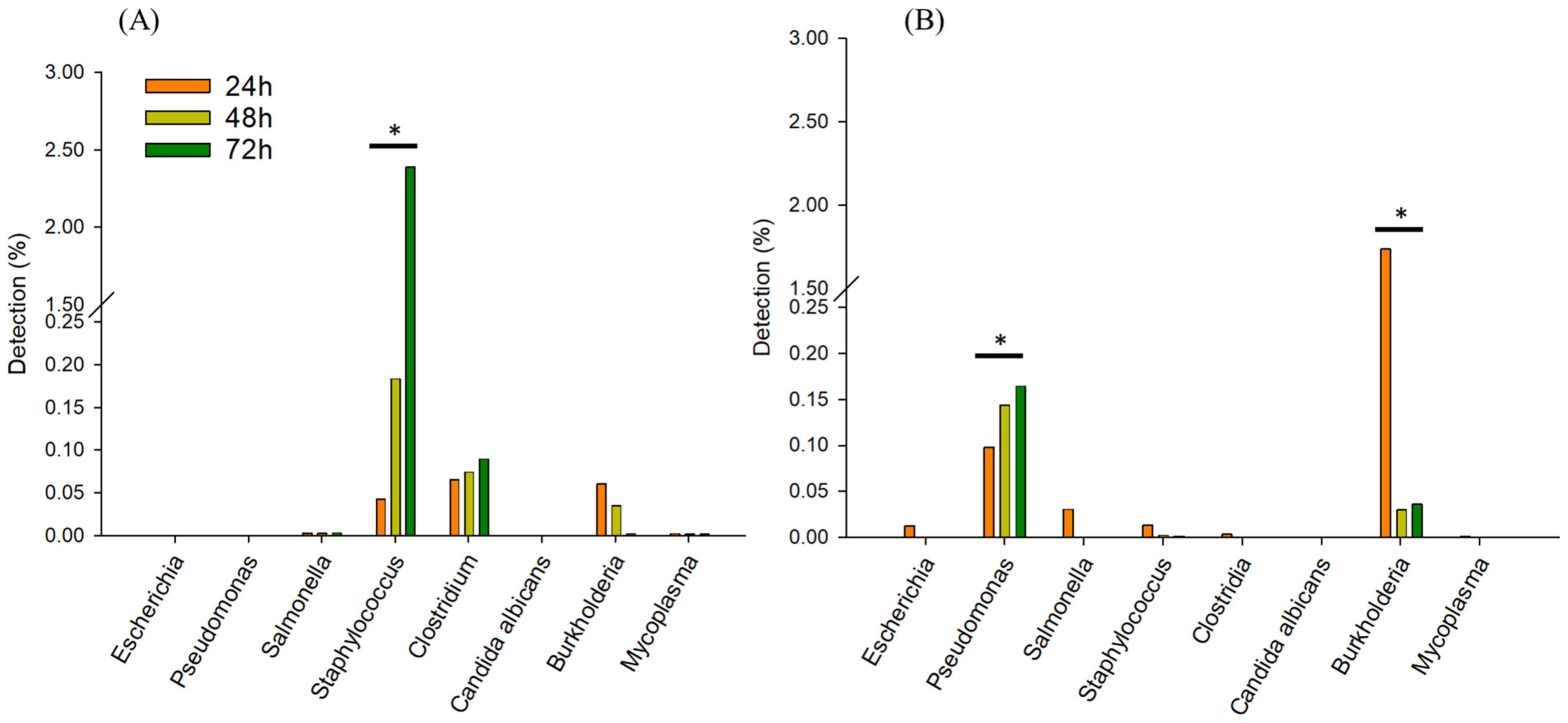
Comparing incubation times; Changes in the relative sequencing read abundance of specified microorganisms in TSB **(A)** and 1/10 strength TSB (1/10 × TSB) **(B)** media at 24 h, 48 h, and 72 h. The * symbol over each diagram indicates a statistically significant difference, *p* < 0.05.

In TSB, only *Staphylococcus* spp. increased from 0.04 (24 h) to 2.3% over 72 h. *Clostridium* spp., was observed similarly between 0.06 to 0.08%, respectively after 72 h (Figure 4A). Interestingly, in 1/10 × TSB, *Pseudomonas* spp. increased from 0.09 (24 h) to 0.16% (72 h) (Figure 4B). However, *Burkholderia* spp. dramatically decreased from 1.7 to 0.03% in 1/10 × TSB. *Salmonella* spp., *Staphylococcus* spp. and *Escherichia* spp. also decreased and were noted to be 0.005% in 1/10 × TSB.

#### 3.2.3 Synergistic effects of BCC-spiked media in detecting “specified microorganisms”

**Figure 5:**
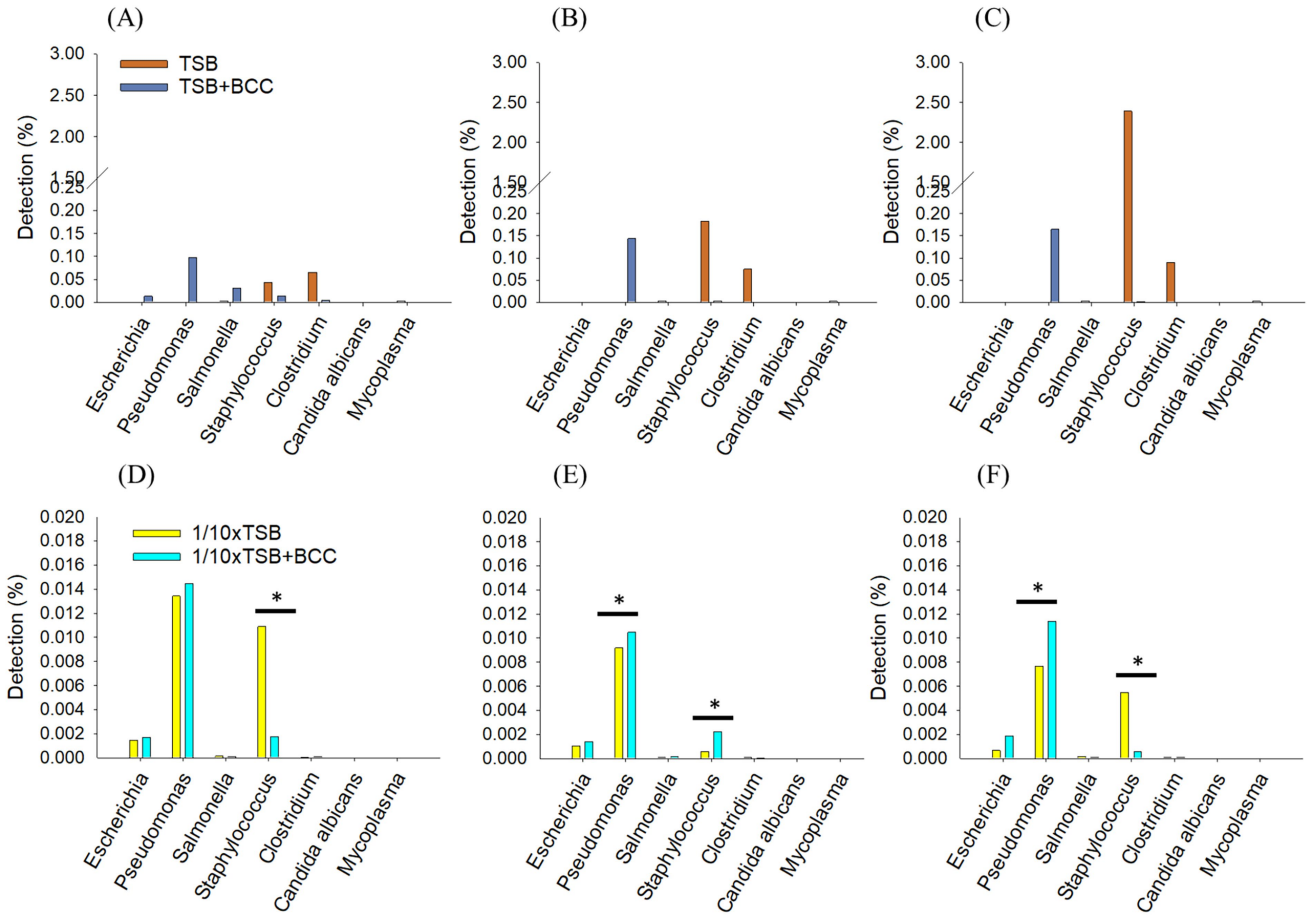
Synergistic effects of BCC-spiked media in detecting specified microorganisms; changes in the relative sequencing read abundance of specified microorganisms in TSB and TSB co-cultured 10^6^ CFU/mL BCC (TSB + BCC) at 24 h **(A)**, 48 h **(B)** and 72 h **(C)** and 1/10 strength TSB (1/10 × TSB) and 1/10 strength TSB co-cultured 10^6^ CFU/mL BCC (1/10 × TSB + BCC) at 24 h **(D)**, 48 h **(E)** and 72 h **(F)**. The * symbol over each diagram indicates a statistically significant difference, *p* < 0.05.

To understand the effect of spiking BCC on the detection of specified microorganisms, we deleted 99% of the BCC read sequences (ranging from 1.1 × 10^7^ reads to 1.9 × 10^7^ reads) in all BCC-spiked media over 72 h. In TSB + BCC, *Pseudomonas* spp. were the most dominant and observed 0.09% at 24 h (Figure 5A). *Salmonella* spp., *Staphylococcus* spp. and *Escherichia* spp. were also observed 0.03, 0.01 and 0.01%, respectively at 24 h. After 48 h and 72 h (Figures 5B,C), *Pseudomonas* spp. increased from 0.14 to 0.16%. However, *Salmonella* spp., *Staphylococcus* spp. and *Escherichia* spp. decreased and were observed less than 0.005%. The main difference between TSB and TSB + BCC is that *Pseudomonas* spp. increased, while *Staphylococcus* spp. and *Clostridium* spp., decreased.

In 1/10 × TSB + BCC, *Pseudomonas* spp., *Staphylococcus* spp., and *Escherichia* spp., were also observed 0.014, 0.002, and 0.001%, respectively at 24 h (Figure 5D). After 48 h and 72 h (Figures 5E,F), *Pseudomonas* spp. were almost constant, from 0.01 to 0.014% over 72 h. *Staphylococcus* spp. and *Escherichia* spp. were observed as being less than 0.05% over 72 h. The difference between 1/10 × TSB and 1/10 × TSB + BCC translates in an increase of *Pseudomonas* spp.

### 3.3 Identifying BCC metabolic pathways in BCC-spiked water

**Figure 6:**
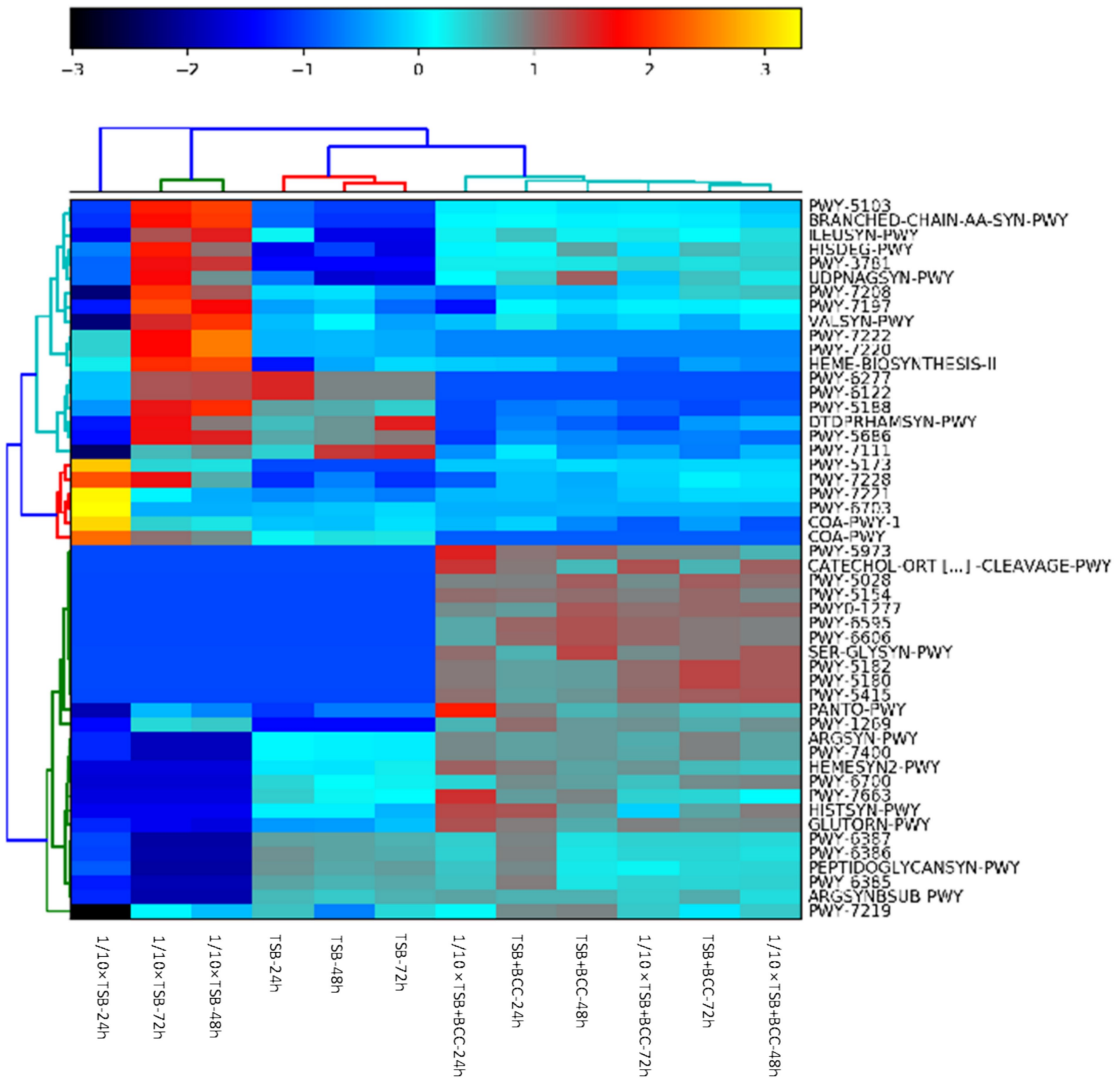
Identification of BCC metabolic pathways in BCC-spiked water. Heatmap of relative abundance of the 50 most abundant metabolic pathways in the metagenomes found in 12 water samples spiked and unspiked with BCC after 24, 48, 72 h enrichment in TSB and 1/10 TSB.

The heatmap shows the clustering of samples and major metabolic pathways identified by whole shotgun metagenome sequencing (Figure 6). A double hierarchical dendrogram was used to analyze metagenome sequences of 12 samples (in different media and incubation times) described in Table 1. Four distinct clusters were identified as observed on the *x*-axis. There was one cluster for TSB + BCC (−24 h, −48 h and −72 h) and 1/10 × TSB + BCC (−24 h, −48 h and −72 h) that stood out as major groups within the samples. The second cluster consisted of TSB (−24 h, −48 h and −72 h). A third cluster consisted of 1/10 × TSB (−48 h and −72 h), yet another cluster represented the remainder of the 1/10 × TSB-24 h. Hierarchical cluster analysis showed that the samples spiked (as co-cultures) with BCC (TSB + BCC and 1/10 strength TSB + BCC) were distinguishable from the unspiked samples (TSB and 1/10 × TSB). As indicated during cluster analysis, the metagenomic data set shows an apparent separation between samples with and without spiking (co-cultures) BCC, indicating a shift in metabolic function when spiked with BCC.

Supplementary Table S2 summarizes the top 20 most abundant metabolic pathways. The most abundant core pathways include preQ0 biosynthesis (PWY-6703) and guanosine ribonucleotides *de novo* biosynthesis (PWY-7221), which were principally found in 1/10 strength TSB-24 h. There is indication that 1/10 × TSB contributes to coenzyme A biosynthesis II (mammalian) (COA-PWY-1) and the superpathway of acetyl-CoA biosynthesis (PWY-5173) pathways. In contrast, the pyruvate fermentation to isobutanol (engineered) (PWY-7111) pathway was dominant in TSB-48 h and TSB-72 h. In BCC-spiked samples (TSB + BCC-24 h and TSB + BCC-48 h), the adenosine ribonucleotides *de novo* biosynthesis (PWY-7219) pathway was principally supported by BCC. Furthermore, phosphopantothenate biosynthesis I (PANTO-PWY), L-ornithine biosynthesis I (GLUTORN-PWY), L-arginine biosynthesis IV (archaebacteria) (PWY-7400), L-arginine biosynthesis I (via L-ornithine) (ARGSYN-PWY), toluene degradation I (aerobic) (via o-cresol) (PWY-5180) and toluene degradation II (aerobic) (via 4-methylcatechol) (PWY-5182) pathways were principally enabled by BCC.

## 4 Discussion

The concern and threat of “specified microorganisms” is not a new public health issue and has been addressed in the general scientific literature as well as in FDA publications. Microbial examination of nonsterile pharmaceutical products is performed according to the methods given in the texts within U.S. Pharmacopeia (USP) <60>, <61>, <62>, <63> and <1,111>. These conventional culture-dependent methods may be limited in detecting specified microorganisms due to their low sensitivity. In this study, metagenomic analysis employing high-throughput sequencing coupled with enrichment using TSB or 1/10 × TSB was able to detect “specified microorganisms” in cold drinking water. The cold drinking water is not used in pharmaceutical production, but these are principles being extrapolated for environmental microbiological assessment. Furthermore, the ability of BCC to stimulate the recovery of specified microorganisms in groundwater enrichments was determined.

Specified microorganisms were observed less than 1.8% relative sequencing read abundance in both TSB and 1/10 × TSB cultures after 24 h incubation. The 1/10 × TSB medium provided a better recovery than TSB on specified microorganisms. Reasoner *et al*. reported that the use of rich culture media, at a relatively high incubation temperature (35°C) and short incubation time (48 h) generally yields lower bacterial counts compared to the use of low nutrient oligotrophic media combined with a low incubation temperature (20°C) and extended incubation times (5–7 days) (Reasoner, 2004). Interestingly, *Burkholderia* spp. (1.73% relative sequencing read abundance) and *Pseudomonas* spp. were predominant in the 1/10 × TSB medium, while *Clostridium* spp., and *Staphylococcus* spp. were predominant in TSB among the specified microorganisms. This result agrees with findings of previous studies that BCC strains were recovered better on diluted TSB than on full strength TSB from distilled water, chlorhexidine gluconate (CHX) and benzalkonium chloride (BZK) solutions (Ahn et al., 2014; Kim et al., 2015). Furthermore, the comparison of TSB and 1/10 × TSB demonstrated that the samples recovered in 1/10 × TSB resulted in higher recovery rates of BCC in distilled water at 6°C, 23°C and 42°C stored for 42 days, than those recovered in TSA (Ahn et al., 2019). Peeters et al. (2016) showed that a 10-fold diluted formulation of *Pseudomonas cepacia* on azelaic acid tryptamine (1/10 strength PCAT) yielded higher diversity compared to PCAT alone. *Burkholderia* spp. and *Pseudomonas* spp. accustomed to living in a low-nutrient aqueous environment may not grow well on rich media. These nutrient-rich media may inhibit or place metabolic stress on BCC populations in oligotrophic nutrient-limited water. Therefore, the nutrient-rich media may favor the growth of faster-growing bacteria (Ahn et al., 2014; Ahn et al., 2019). Concordantly, Peeters et al. (2016) showed that a pre-enrichment with 0.1% peptone yielded 36.8% increased recovery of *Stenotrophomonas maltophilia* from water samples. In our study, *Bacillus* spp. (97–99% relative sequencing read abundance) and *Stenotrophomonas* spp. (61.5–97% relative sequencing read abundance) were dominant in TSB and 1/10 × TSB, respectively. Although TSA and TSB are generally considered media of choice, technical specifications for USP surveys of specified aerobic bacteria from non-sterile pharmaceutical materials allow for different oligotrophic media to be used.

Incubation conditions were required for the microbiological examination of non-sterile products in USP (USP60, 2018; USP61, 2016; USP62, 2016). Traditional methods depend on the ability of microorganisms to yield visible colonies after an incubation period of ≤3–5 days at 30–35°C (USP61, 2016). It is well documented that the recovery of bacteria can vary by 10 to 30% depending upon the choice of medium and incubation conditions (Wilson and Varney, 1995; Reasoner, 2004). In this study, we evaluated the influence of incubation time on metagenomics analysis efficiency for detecting specified microorganisms as well as the benefits that could be achieved with extended incubation times. Meder et al. (2012) reported that Milliflex Quantum detection of microorganisms was achieved after 9 h and 12 h of minimal incubation time with TSB for *Bacillus subtilis* and *Staphylococcus aureus*, respectively. *Pseudomonas aeruginosa* and *Candida albicans* needed between 16 h and 22 h incubation times. Most clinical pathogens grow easily over a 24–48 h time period on solid agar media, but several bacterial species are slow growing, requiring extended incubation time, from 3 to >45 days (Lagier et al., 2015). *S. aureus, P. aeruginosa*, and *B. subtilis* were detected by the Rapid Milliflex Detection System (RMDS) within 5 days (Parveen et al., 2011). In this study, pre-enrichment prior to metagenomics analysis demonstrated specified microorganisms could be detected in aqueous samples after 24 h of incubation, compared to 3 days required by the traditional culture-dependent method. Our study showed that an incubation period of 24 h is sufficient for detecting specified microorganisms by metagenomics analysis.

Microbial populations in most natural environments consist of a multitude of species, which interact with one another to acquire the nutrients necessary for them to survive. This implies that bacteria compete with their neighbors for space and resources resulting in the disruption of microbial communities (Hibbing et al., 2010). We observed that spiking water samples with BCC can enhance or diminish recovery of specified microorganisms. The results of this study show that inoculation with BCC yielded more benefits in recovering *Pseudomonas* spp. compared to uninoculated controls in 1/10 × TSB. *Pseudomonas* spp. (*Gammaproteobacteria*) and BCC (*Betaproteobacteria*) are found in the same type of soil as well as in lungs of cystic fibrosis (CF) patients (Weaver and Kolter, 2004). Relatively little is known at the molecular level about how different bacteria interact with each other while coexisting in a particular environment (Agnoli et al., 2019). One of the most widely studied systems is that of siderophores (e.g., ornibactin and pyochelin), high-affinity iron chelating compounds produced by microorganisms such as BCC, which can be secreted by *P. aeruginosa* spp. (Weaver and Kolter, 2004; Agnoli et al., 2019). In an environment with low iron levels, sufficiently high intracellular iron concentrations are critical for bacterial survival. When iron levels are low, BCC secrete chemically diverse siderophores to capture ferric ions (Fe^3+^). The synthesis of the two main siderophores, ornibactin and pyochelin, is regulated in an iron concentration dependent manner via the regulator protein. In fact, studies have shown that in the co-culture with *B. cepacia*, *P. aeruginosa* can detect and respond to ornibactin produced by *B. cepacia* via the production of xenosiderophores in order to sequester iron for itself (Weaver and Kolter, 2004; Hibbing et al., 2010). Therefore, *P. aeruginosa* can easily obtain the necessary iron by collecting it from other organisms while precluding other microorganisms from acquiring iron (Scott et al., 2019). In co-culturing of BCC strain BC-7/J-1 and *P. aeruginosa* Pae33 or of BCC strain BC-7/J-1 and *P. aeruginosa* PAO1 (equal numbers or a 100:1 ratio), however, BCC strains were killed after 48 h by *P. aeruginosa* via the secretion of antibacterial product that was toxic to BCC bacteria rather than simple substrate competition (Schwab et al., 2014). Subsequently, an additional study has shown that *P. aeruginosa* can effectively kill BCC *in vitro*, for which hydrogen cyanide (HCN) was recently proposed to play a critical role (Bernier et al., 2016). In this study, no evidence was found to account for a higher recovery of *Pseudomonas* spp. Nevertheless, a higher number of *Pseudomonas* spp. was obtained from the TSB and 1/10 × TSB media spiked with BCC, compared to the media without BCC. Future work will be required to examine *P. aeruginosa* genes that are induced in presence of BCC to understand how these two species interact with each other. To mitigate the overrepresentation of *Burkholderia cepacia* complex, alternative library preparation methods or sequencing strategies should be considered.

Taxonomic groups in a sample may indicate specific functions and ecological roles, such as the synergistic relationships between oligotrophic bacteria and BCC, highlighting the importance of integrating taxonomic and functional analyses to better understand microbial community dynamics and their interactions with the environment. This taxa information can be linked with metabolic pathways, which could be ultimately used to elucidate functional roles of microbiomes. We found a higher functional capacity for toluene degradation (PWY-5180 and PWY-5182) in BCC-spiked (co-cultures) samples, suggesting BCC could contribute to aerobic toluene degradation. These discriminative metabolic pathways were present in all BCC-spiked (co-cultures) samples. Hence, functional perspectives of these pathways require further investigation by integrating metatranscriptomics and metabolomics. *Burkholderia* spp. are known to grow in presence of benzene, toluene, ethylbenzene, benzoate, catechol, or trichloroethylene used as carbon and energy sources (Mars et al., 1996; Johnson and Olsen, 1997; Hamid et al., 2014; Lunsmann et al., 2016). Furthermore, the MetaCyc database of metabolic pathways for aerobic toluene degradation via *o*-cresol (PWY-5180) was targeted to assess predicted capacity for aerobic toluene degradation [MetaCyc toluene degradation I (aerobic) (via o-cresol)]. In contrast to our previous findings using filtration to concentrate cells followed by DNA extraction and subsequent metagenomics analysis, carbohydrates based level 1, clustering-based, and amino acids and derivatives subsystems were the most abundant level 1 subsystem in water collected from a cold water fountain in Hot Springs, AR (Daddy Gaoh et al., 2024). In this regard, metagenomic analysis of BCC-spiked samples has provided new insights into metabolic pathways in water samples and the difference between cultured water samples vs. BCC-spiked samples. The presence of PWY-5180 and PWY-5182 is important since they can be used as BCC indicators under different environmental conditions.

We have previously detected members of specified microorganisms using metagenomics analysis of water samples collected from a cold water fountain in Hot Springs, AR, although it was difficult to correlate their prevalence to rates or extents of relative sequencing read abundance (Daddy Gaoh et al., 2024). It is important to remember that microbial populations in most natural environments consist of a multitude of species and little is known about how different bacterial species may affect each other in a given environment. Furthermore, over 99% of the microorganisms present in many natural environments are not readily culturable, making it challenging to correlate data from pre-enrichment and non-enriched samples (Streit and Schmitz, 2004). Compared to the 0.1–18.3% previously reported in unenriched samples (Daddy Gaoh et al., 2024), specific species were found in less than 2% of the total reads in this study. This highlights the complexity of using pre-enrichment with metagenomics analysis in surveying microbial populations. In fact, findings by Jarvis et al. (2015) on cilantro microbiome before and after nonselective pre-enrichment for *Salmonella* using 16S rRNA and metagenomic sequencing showed that uncultured samples had an abundance of *Proteobacteria* at time zero, while the 24-h enriched samples were mostly composed of Gram-positive *Firmicutes*. Another finding by Ottesen et al. (2013) shows that the enrichment process can co-enrich non-target organisms, potentially inhibiting or even killing the target pathogen. Additionally, the potential presence of bacteriophages in samples can also affect enrichment cultures by infecting and lysing target bacteria, reducing their numbers and altering the isolated biotypes (Muniesa et al., 2005). Consequently, enrichment of environmental samples can significantly alter the taxonomic profile and may not increase the probability of detecting target pathogens, thus resulting in the creation of biased samples, ultimately leading to different taxonomic profiles and abundances compared to unenriched samples (Pettengill et al., 2012). These findings emphasize the need to develop and validate culture-independent metagenomic methods for pathogen detection in complex environmental samples. Another area that could be developed resides in the determination of viability or infectious potential of detected microorganisms. While high-throughput DNA sequencing-based techniques have been quite useful for determining the composition of microbial communities in various environments, current DNA metagenomic sequencing technologies are unable to distinguish between viable and dead cells. To address this conundrum, propidium monoazide (PMA) treatment can be applied to selectively remove DNA from dead cells before sequencing (Tantikachornkiat et al., 2016; Daddy Gaoh et al., 2022a; Yap et al., 2022; Daddy Gaoh et al., 2023). Recently, Liu et al. (2021) used a combination of PMAxx (an enhanced form of propidium monoazide [PMA]) treatment and metagenomic sequence extraction, to successfully distinguish between live and dead bacteria in human saliva and feces samples. These methods can be combined with high-throughput sequencing technologies to provide insights into community composition, metabolic potential, and physiological states of microorganisms (Liu et al., 2021; Yap et al., 2022). While promising, these techniques require further optimization of PMAxx / PMA treatment for complex environmental samples to capture the full diversity of microbial life forms and metabolic states. As next-generation sequencing technologies improve and the costs continue to drop, recent technical improvements geared at very low concentrations can lead to effectively discriminating viable cells from dead cells.

Finally, it is important to emphasize the sensitivity-related challenges associated with metagenomic approaches. While traditional culture methods typically require 10^2^–10^3^ CFU/mL or more for reliable detection and are limited to organisms that can grow under specific conditions, PCR-based techniques offer greater sensitivity. Quantitative PCR (qPCR) can detect approximately 10^1^–10^2^ genome copies per reaction, although its accuracy may be affected by the presence of inhibitors in complex matrices. Droplet digital PCR (ddPCR) improves upon this, often detecting as few as 1–10 copies per reaction due to its partitioning and absolute quantification capabilities (Ahn et al., 2020). In contrast, shotgun metagenomic sequencing generally has a higher limit of detection around 10^3^–10^4^copies/mL, depending on factors such as sequencing depth, DNA extraction efficiency, and microbial background. Although, enrichment strategies have been shown to improve detection sensitivity, they introduce certain limitations. Culture-enriched phenotypic metagenomics has recently demonstrated its utility in the detailed and sensitive characterization of beta-lactam resistomes in wastewater and surface water environments (Zhang et al., 2022). Similarly, shotgun sequencing of culture-enriched samples has enhanced the identification of critical antimicrobial resistance (AMR) genes in surface waters, as part of the U.S. National Antimicrobial Resistance Monitoring System (NARMS) (Ottesen et al., 2022; Franklin et al., 2024). However, a significant trade-off associated with the use of enrichment techniques is the loss of the original quantitative context of the sample, which can hinder the interpretation of microbial abundance and dynamics (Davis et al., 2025). Furthermore, metagenomic analysis should be considered of the detection limits (*i.e.*, greater than 10^6^ bacteria per gram of feces) (Lagier et al., 2015), and rare species with low abundance can be missed (Aardal et al., 2024). Oligotrophic enrichment utilizing shotgun metagenomic sequencing can facilitate the identification of specific organisms in pharmaceutical water and other non-sterile pharmaceutical products. To improve the detection of specified microorganisms in enriched groundwater, it would be beneficial to concentrate processes using larger volumes of groundwater and to validate the detection limits using reference microorganisms. However, additional research is required, including the incorporation of an internal reference standard, the development of a standardized protocol for sample preparation, as well as the establishment of bioinformatic thresholds and benchmarking toward PCR-based methods. These steps are essential for the broader implementation of the combined enrichment method with metagenomic analysis for the detection of specific organisms in non-sterile pharmaceutical products.

## 5 Conclusion

Using the whole shotgun metagenomic sequencing, we identified *Bacillus* spp. and *Stenotrophomonas* spp. to be the predominant species detected when samples were enriched in TSB and 1/10 strength TSB, respectively. The biodiversity of TSB-enriched samples was lower than that of 1/10 strength TSB, underscoring the significance of choosing the right enrichment techniques for the identification of particular bacterial targets. According to our results, *Burkholderia* spp., *Pseudomonas* spp., *Salmonella* spp., and *Escherichia* spp. were primarily found in 1/10 × TSB, whereas *Clostridium* spp. and *Staphylococcus* spp. were primarily found in TSB-enriched samples. Our findings also revealed, five MetaCyc pathways, including toluene degradation I (aerobic) (via o-cresol) (PWY-5180) and toluene degradation II (aerobic) (via 4-methylcatechol) (PWY-5182), which were discriminative across groups in BCC-spiked samples. Consequently, more studies are needed to understand these pathways in diverse water samples for their potential use as BCC indicators. Our shotgun metagenomic sequencing approach may be used for a better detection of specified microorganisms in non-sterilized pharmaceutical products, which were previously missed or not identified by traditional culture-dependent methods. Future work will focus on quantifying viable microbes, develop and validate culture-independent metagenomic methods for both unprocessed and processed water used in pharmaceutical manufacturing.

## Data Availability

Metagenomic data deposited to NCBI with accession number PRJNA1301903.

## Footnotes

^1^ http://www.mrdnalab.com

^2^ http://basespace.illumina.com

^3^ https://nephele.niaid.nih.gov
