# Supplementary material for: A metagenomic analysis coupled with oligotrophic enrichment approach for detecting specified microorganisms in potable groundwater samples
Source: Front Microbiol. 2025 Aug 13;16:1645324. doi: 10.3389/fmicb.2025.1645324 (PMC12382351; doi:10.3389/fmicb.2025.1645324)

**Supplementary Figure S2**

Changes at the genus level in the relative abundance in cold fountain water. In TSB and 1/10× TSB medium with or without BCC from 24 h (A), 48 h (B), and 72 h (C), sequences were generally dominated by one genus. TSB + BCC, TSB inoculated with 10^6^ CFU/mL of BCC; 1/10× TSB, 1/10 strength TSB; 1/10× TSB + BCC, 1/10× TSB inoculated with 10^6^ CFU/mL of BCC.


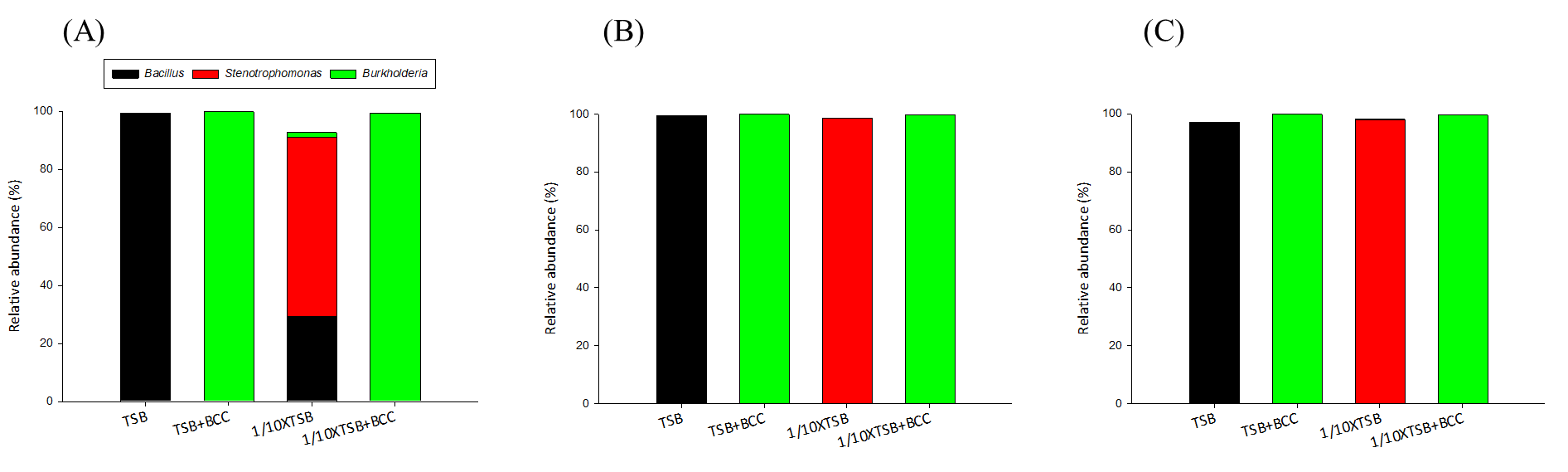

Supplement: Supplementary file 1 [file Supplementary_file_1.zip › Supplementary Figure 2.DOCX]
