# Supplementary material for: A metagenomic analysis coupled with oligotrophic enrichment approach for detecting specified microorganisms in potable groundwater samples
Source: Front Microbiol. 2025 Aug 13;16:1645324. doi: 10.3389/fmicb.2025.1645324 (PMC12382351; doi:10.3389/fmicb.2025.1645324)

**Supplementary Figure S1** FastQC provide quality control checks on raw sequence data coming from high throughput sequencing pipelines.

.


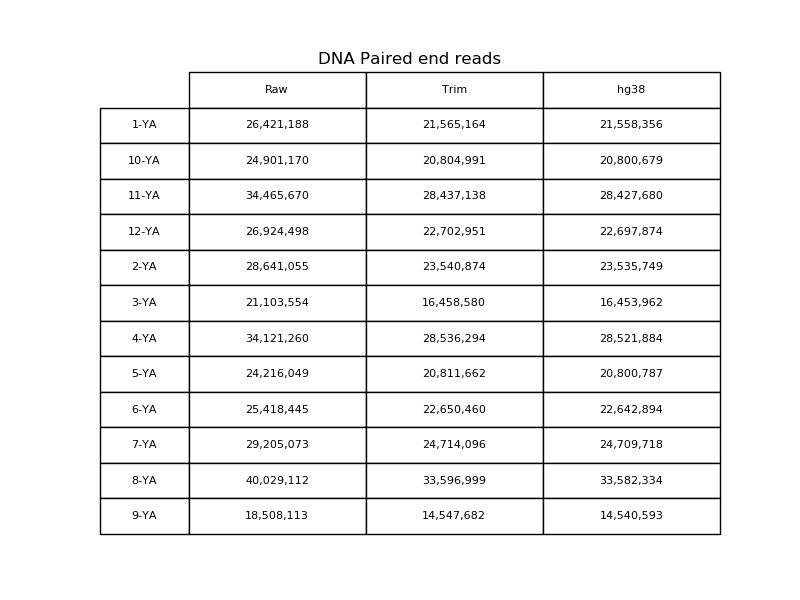


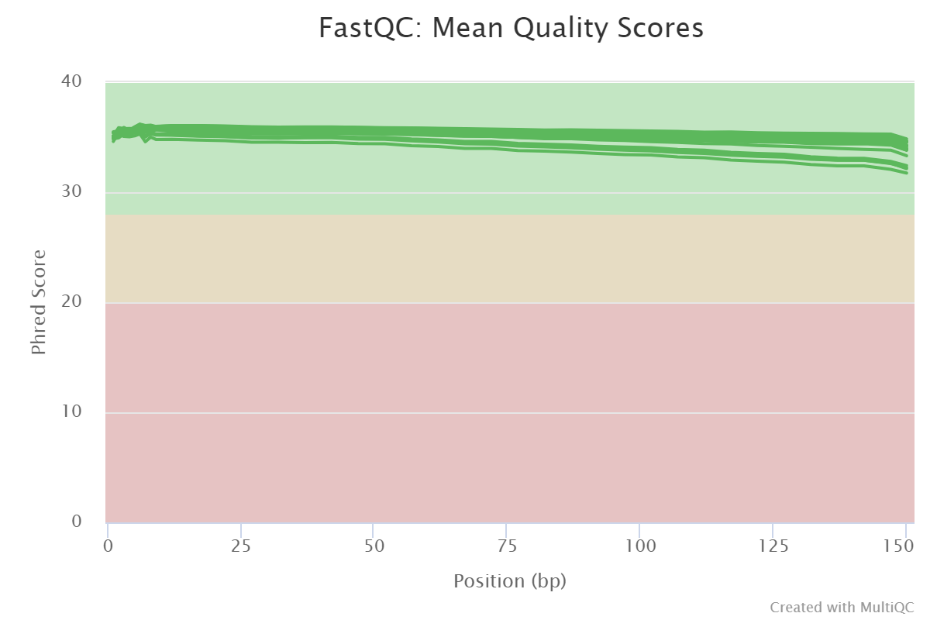


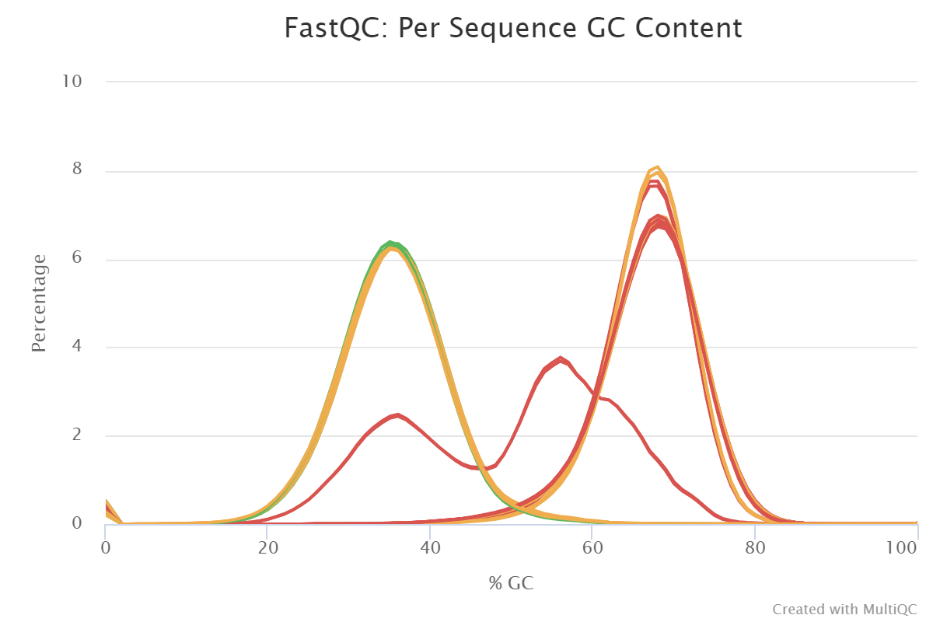


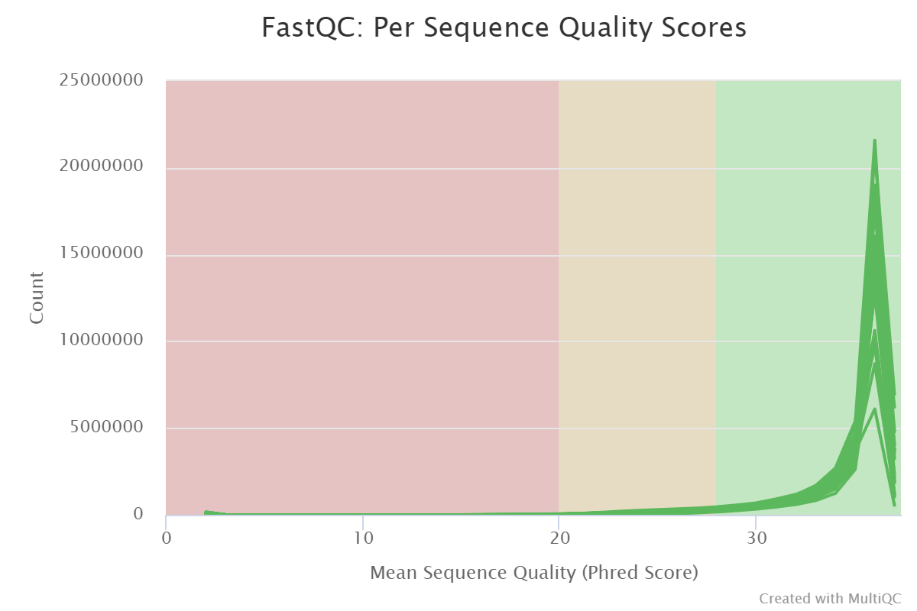


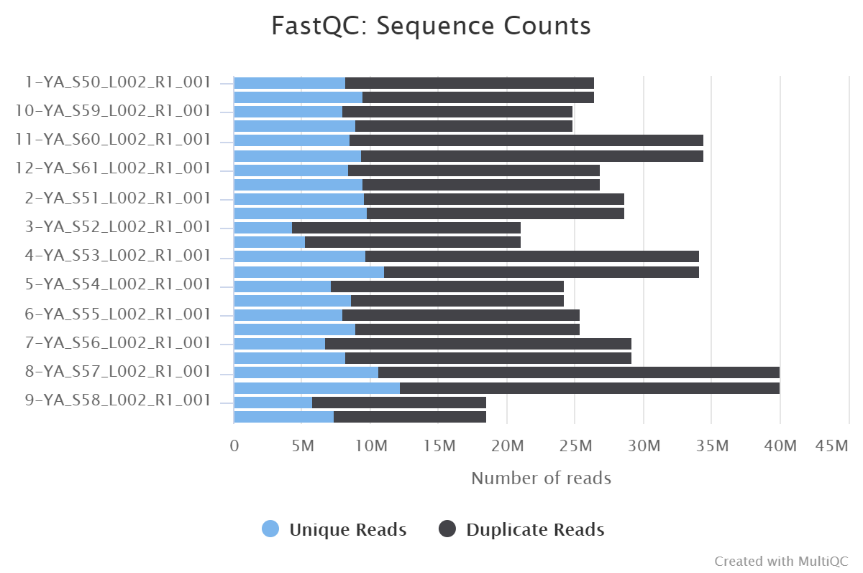


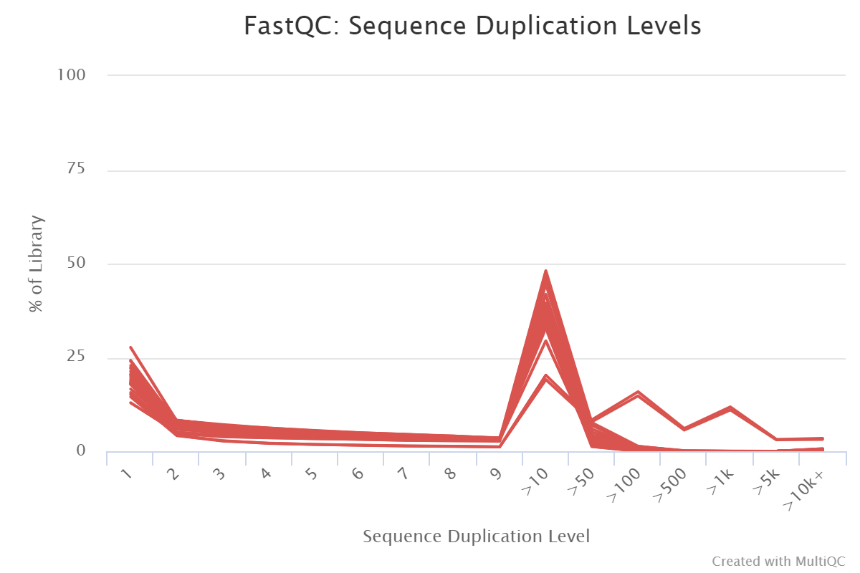


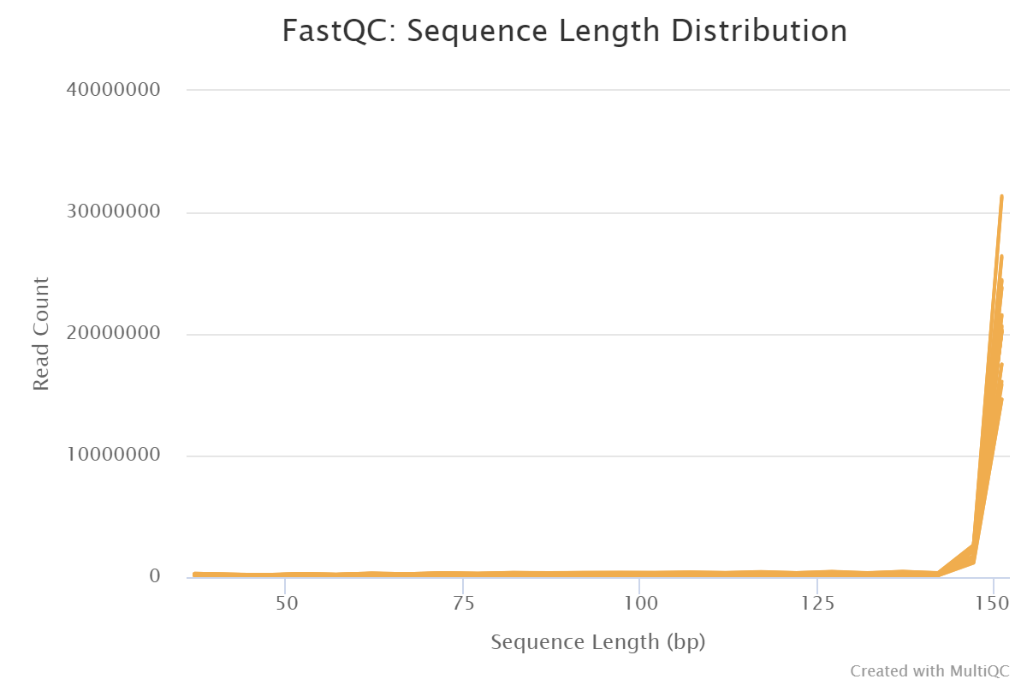

Supplement: Supplementary file 1 [file Supplementary_file_1.zip › Supplementary Figure 1.DOCX]
