# Supplementary material for: A metagenomic analysis coupled with oligotrophic enrichment approach for detecting specified microorganisms in potable groundwater samples
Source: Front Microbiol. 2025 Aug 13;16:1645324. doi: 10.3389/fmicb.2025.1645324 (PMC12382351; doi:10.3389/fmicb.2025.1645324)
Supplement: Supplementary file 1 [file Supplementary_file_1.zip › Supplementary Table 2.DOCX]

**Supplementary Table S2.** Top 20 pathways by average abundance (partial table). Bold is BCC-spiked (co-culture) samples (TSB + BCC-24 h and TSB+BCC-48 h)

| Pathways | | Average | Variance |
| --- | --- | --- | --- |
| **1** | **PWY-5182: toluene degradation II (aerobic) (via 4-methylcatechol)** | **0.0135** | **0.000187** |
| **2** | **PWY-5180: toluene degradation I (aerobic) (via o-cresol)** | **0.0135** | **0.000187** |
| 3 | ILEUSYN-PWY: L-isoleucine biosynthesis I (from threonine) | 0.0139 | 7.72E-05 |
| **4** | **ARGSYN-PWY: L-arginine biosynthesis I (via L-ornithine)** | **0.0141** | **5.77E-05** |
| **5** | **PWY-7400: L-arginine biosynthesis IV (archaebacteria)** | **0.0141** | **5.79E-05** |
| 6 | PWY-7208: superpathway of pyrimidine nucleobases salvage | 0.0143 | 1.60E-05 |
| 7 | PWY-7197: pyrimidine deoxyribonucleotide phosphorylation | 0.0145 | 1.28E-05 |
| 8 | PWY-5173: superpathway of acetyl-CoA biosynthesis | 0.0149 | 0.00023 |
| **9** | **GLUTORN-PWY: L-ornithine biosynthesis I** | **0.015** | **7.85E-05** |
| 10 | HISDEG-PWY: L-histidine degradation I | 0.0152 | 1.73E-05 |
| 11 | PWY-7228: superpathway of guanosine nucleotides de novo biosynthesis I | 0.0154 | 2.50E-05 |
| 12 | COA-PWY-1: coenzyme A biosynthesis II (mammalian) | 0.0155 | 3.18E-05 |
| 13 | PWY-3781: aerobic respiration I (cytochrome c) | 0.0155 | 0.00012 |
| 14 | UDPNAGSYN-PWY: UDP-N-acetyl-D-glucosamine biosynthesis I | 0.0167 | 7.90E-05 |
| 15 | VALSYN-PWY: L-valine biosynthesis | 0.0171 | 2.59E-05 |
| **16** | **PANTO-PWY: phosphopantothenate biosynthesis I** | **0.0182** | **3.51E-05** |
| 17 | PWY-7111: pyruvate fermentation to isobutanol (engineered) | 0.0184 | 2.47E-05 |
| **18** | **PWY-7219: adenosine ribonucleotides de novo biosynthesis** | **0.0185** | **1.71E-05** |
| 19 | PWY-7221: guanosine ribonucleotides de novo biosynthesis | 0.0186 | 0.000139 |
| 20 | PWY-6703: preQ0 biosynthesis | 0.0576 | 0.0135 |
